# Muscle Activity After Stroke: Perspectives on Deploying Surface Electromyography in Acute Care

**DOI:** 10.3389/fneur.2020.576757

**Published:** 2020-09-23

**Authors:** Katherine M. Steele, Christina Papazian, Heather A. Feldner

**Affiliations:** ^1^Department of Mechanical Engineering, University of Washington, Seattle, WA, United States; ^2^Department of Rehabilitation Medicine, University of Washington, Seattle, WA, United States

**Keywords:** electromyography, translation, stroke, arm, paralysis, data visualization

## Abstract

After a stroke, clinicians and patients struggle to determine if and when muscle activity and movement will return. Surface electromyography (EMG) provides a non-invasive window into the nervous system that can be used to monitor muscle activity, but is rarely used in acute care. In this perspective paper, we share our experiences deploying EMG in the clinic to monitor stroke survivors. Our experiences have demonstrated that deploying EMG in acute care is both feasible and useful. We found that current technology can be used to comfortably and non-obtrusively monitor muscle activity, even for patients with no detectable muscle activity by traditional clinical assessments. Monitoring with EMG may help clinicians quantify muscle activity, track recovery, and inform rehabilitation. With further research, we perceive opportunities in using EMG to inform prognosis, enable biofeedback training, and provide metrics necessary for supporting and justifying care. To leverage these opportunities, we have identified important technical challenges and clinical barriers that need to be addressed. Affordable wireless EMG system that can provide high-quality data with comfortable, secure interfaces that can be worn for extended periods are needed. Data from these systems need to be quickly and automatically processed to create round-ready results that can be easily interpreted and used by the clinical team. We believe these challenges can be addressed by integrating and improving current methods and technology. Deploying EMG in the clinic can open new pathways to understanding and improving muscle activity and recovery for individuals with neurologic injury in acute care and beyond.

## Introduction

Every brain injury is unique—making individualized evaluations especially important for diagnosis and prognosis. For individuals who have had a stroke, impaired movement is one of the most persistent and disabling sequela, severely limiting participation, and quality of life (1–3). Many individuals initially have limited or no ability to move their limbs after stroke. However, determining when and if an individual will regain movement is challenging (4–6). Surface electromyography (EMG) provides a non-invasive window to observe neuromotor activity. By monitoring activity and observing resulting movements, we can evaluate the integrity of neuromotor pathways (7). The initial weeks after stroke are viewed as a critical period of neural plasticity and recovery (8), yet EMG is rarely deployed during this time.

In acute care, function-based clinical exams remain the standard for evaluating and monitoring muscle activity and movement. The Manual Muscle Test (MMT) and NIH Stroke Scale (NIHSS) are among the most common evaluation measures used in the United States. These measures are often performed daily in the hospital to track recovery and document outcomes for insurance purposes. Clinicians conduct these measures by asking individuals to attempt to voluntarily move specific body parts, assigning an ordinal score based upon observed movement or muscle activity felt by palpation (9–12). Members of the care team can conduct these exams quickly, but they are coarse measures that provide limited insight into the extent of injury or prognosis, especially for individuals with language barriers, receptive aphasia, neglect, or other impairments that limit ability to follow instructions. The Fugl-Meyer Assessment (FMA) expands the repertoire of movements to evaluate synergistic or other inappropriate muscle activity (13, 14). While the FMA has shown promise for predicting recovery and future function (15), it is not often used in the clinic due to the time and training required. Like the MMT and NIH Stroke Scale, it also has limited utility for individuals with impaired voluntary movement or difficulty following instructions. An ideal assessment tool to monitor muscle activity and movement after stroke would provide deeper insight into the quantity and quality of movement, while requiring minimal time to execute.

In the 1950s, clinicians like Thomas Twitchell deployed EMG to monitor muscle activity (16–19), but today EMG is mainly confined to research settings. Twitchell's detailed observations of EMG recordings from stroke survivors in acute care remain some of our most detailed descriptions of early muscle activity after stroke. Twitchell would not recognize today's sophisticated EMG systems (20). Large sensors and tangles of wires have been replaced by sleek, small packages that wirelessly transmit data from dry electrodes that make it easier to target and isolate activity from individual muscles. Material selection and electrode design continue to improve, such that EMG sensors can even be worn for multiple days with minimal impact on signal quality or skin health (21–23). One of the largest changes has come in our processing and analytic ability. We have replaced the chart recorders that Twitchell used with systems that easily capture and analyze recordings (24, 25). EMG sensors can also be integrated with other sensors, such as inertial measurement units (IMUs) that provide concurrent measurements of movement.

Despite all of the opportunities provided by this advancement, the translation of EMG to clinical care has been a slow process. In this paper, we share our team's perspective translating EMG into the clinic through a multidisciplinary collaboration between engineers and clinicians. Over the past two years, we have monitored muscle activity with adult stroke survivors within the first 5 days after stroke. This experience has shown our team that there are great opportunities in expanding the use of EMG in clinical care, but significant barriers that need to be overcome to facilitate this translation. We hope our experiences and lessons learned can support other teams attempting this translation and accelerate the use of EMG technology to advance care.

## Surface EMG In Acute Care

“I think my finger moved today” is a phrase that many clinicians in acute stroke care or rehabilitation have heard from a stroke survivor. During the early weeks, movement can return rapidly and seemingly unexpectedly, which makes every twitch or sensation a potential positive sign (19). Clinicians and their patients often cannot definitively determine whether an individual voluntarily moved their arm or finger, or if there were changes compared to yesterday (26, 27). While a clinician cannot wait by the bedside, an EMG system can unobtrusively monitor muscle activity while the patient and clinical team continue with standard care. Of course, the acute setting presents unique challenges in deploying any technology (28). Large care teams work around the clock to coordinate and conduct numerous tests and procedures to address the initial injury and prevent further damage.

**Figure 1:**
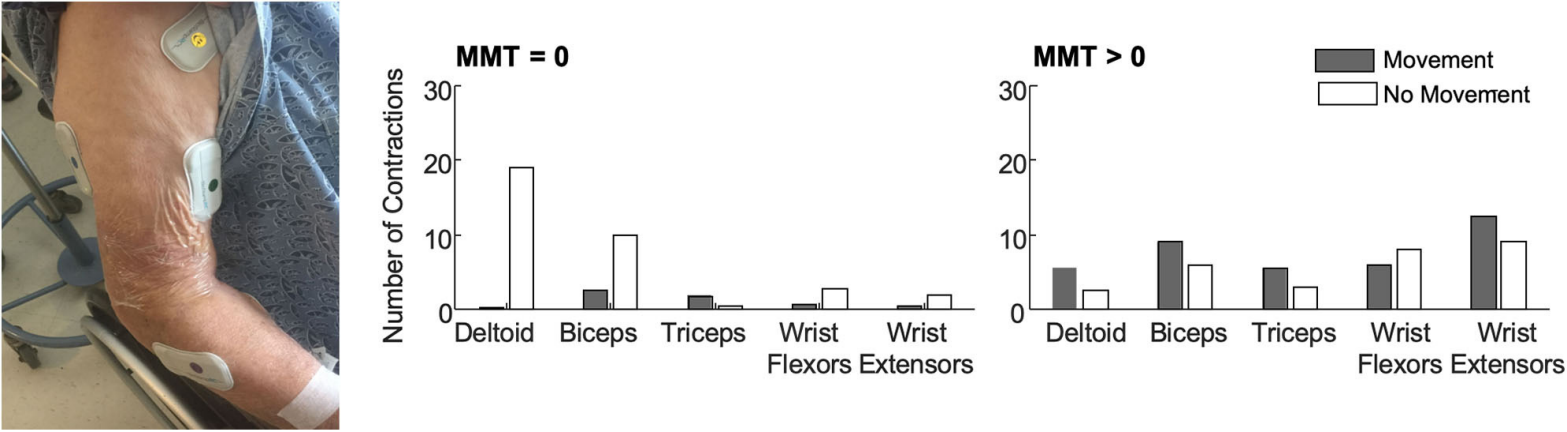
**(Left)** BioStamp sensors provided a wireless and low-profile sensor to monitor muscle activity. We monitored muscle activity from five muscle groups on the paretic arm—the deltoid, biceps, triceps, wrist extensors, and wrist flexors. Tegaderm and Coband were placed over the electrodes to ensure they did not fall off or get stuck to bed sheets during 4 h of monitoring. The EMG data were used to evaluate outcome metrics like the median number of contractions (per 30-min of analyzed data) among patients with no observable muscle activity (*N* = 11, MMT = 0) and patients with some residual muscle activity (*N* = 10, MMT > 0). Accelerometer data were used to classify each contraction as occurring during periods with or without movement. **(Right)** Median number of contractions identified for each muscle with and without movement. Importantly, contractions were identified for all five muscles in all patients. For participants with MMT > 0, contractions were identified in all five muscles in a single 30-min monitoring session. Up to 3 h of monitoring was required to detect contractions in all five muscles for the participants with MMT = 0. As expected, participants with MMT > 0 had more contractions with movement. For participants with MMT = 0, contractions during movement likely reflect times when their arm was being moved during care. Participants with MMT = 0 also had more contractions in proximal muscle groups.

In our work to deploy EMG in this challenging environment, our team prioritized selecting an EMG system that provided wireless sensing in a compact form. The BioStampRC sensors (BioStampRC, MC10, Lexington, MA) included integrated EMG and accelerometer sensors that could concurrently monitor muscle activity and movement. We targeted the muscles most commonly assessed by the clinical team, placing sensors on five muscle groups: the deltoid, biceps, triceps, wrist flexors, and wrist extensors of the affected upper extremity (Figure 1). We followed SENIAM guidelines for placing the sensors, but often had to adjust to accommodate IV's, bandages, or telemetry pads. Loose skin, adipose tissue, and sweat were also common issues that impacted signal quality and sensor adherence.

We deployed these sensors with stroke survivors who demonstrated impaired arm movement (NIHSS > 1) at a level-one trauma hospital. Patients were excluded if they were on comfort care, but otherwise we had broad inclusion as our main goal was evaluating deployment of the technology and observing muscle activity of all stroke survivors. We recruited patients from the acute stroke unit, where some patients may have received initial care in the intensive care unit. At this hospital, most stroke survivors stay in acute care for <2 weeks, receiving daily evaluations and therapy, before being discharged to inpatient rehabilitation, a skilled nursing facility, or their home. Our primary objective was to evaluate whether muscle activity could be detected during acute stroke care. We were especially interested in determining whether EMG sensors could detect muscle activity for those patients classified as having dense hemiplegia or flaccidity, who could not participate or be evaluated with other clinical measures. For each patient, we collected up to four hours of data. We manually identified contractions for each muscle, marking the start and stop time and coding each contraction as during periods of movement or rest based upon concurrent accelerometer data. Details on the data collection, EMG processing, and analyses can be found in (29, 30) and (REF), while here we aim to share key experiences in deploying this technology.

For the patients we monitored, muscle contractions were detected from all five muscles during a single four hour collection period during standard care (Figure 1). This was true even for the patients who had an MMT score of zero (*N* = 11), indicating no voluntary movement or muscle activity detected via palpation. For the participants with an MMT >0 (*N* = 10), only a single 30-min time window was required to identify contractions in all five muscles. For the patients who were initially flaccid, we did find moderate correlations between early contraction characteristics and scores on the MMT at follow-up. These findings indicate that muscle activity is present during the first week after stroke, even among participants characterized as flaccid, and EMG can provide quantitative metrics that may have prognostic value for predicting future function.

## Lessons Learned

Our experiences conducting this research presented several important lessons to inform translation of EMG into stroke rehabilitation. These lessons reflect the clinical realities of working in acute care, as well as opportunities to enhance care by using EMG for monitoring, diagnosis, or biofeedback. There are numerous technical and logistical hurdles that need to be overcome to take advantage of these opportunities—from sensor design to automated processing to clinician education. Deploying new technology in the clinic requires concerted and collaborative efforts, but can open new pathways for improving care and recovery.

### Lesson 1: From Data to Unique Insights

While EMG provides compelling, quantitative metrics to monitor function and recovery, translating this technology into the clinic requires these data provide unique and compelling insights. There are numerous ways we believe EMG could enhance acute stroke care. Foremost, EMG may be useful for monitoring—allowing patients, families, and the care team to view daily updates on changes in muscle activity and movement. While the MMT can provide information about large-scale changes, the subtle changes we observed using EMG can be important for supporting patient motivation, providing evidence for discharge decisions (e.g., is progress being observed?), and determining eligibility for inpatient rehabilitation or other services.

Beyond monitoring, our clinical team and participants also suggested that real-time EMG data may be useful for biofeedback applications (31–33). Clinicians may use these data to enhance or supplement their clinical exams. Measures like the MMT rely on the clinician using palpation to try to detect muscle activity. If an EMG sensor was already on, the clinician could look at the live feed to quickly view and validate their observations. If a patient was struggling to understand the clinician's instructions, the EMG data could also be used to help them understand the desired action. These additions could improve the repeatability of these exams, which often have poor inter-rater and inter-session repeatability (9, 34). As one physical therapist imagined (35):

“If I can't get them to do a certain movement, I'm like, ‘Well, is there any activity in that muscle?' That would be helpful to get that information in terms of assessing, ‘Oh, yeah, there's a little bit here', and then a couple of sessions later, ‘Hey, there's a lot more activity.”

Another observation from this work was that there is a lot of downtime for most patients in acute care (36, 37). EMG sensors that are being used for monitoring could serve double-duty by providing early opportunities to practice. A simple display, on the television or a mobile application, could let patients view their muscle activity, control devices (e.g., change the channel), or play simple games controlled by EMG signals.

EMG may also be useful for diagnosis or prognosis. Contraction characteristics from EMG could complement imaging to help neurologists decipher the extent of neural damage (38–40). While two insults may appear similar, EMG recordings may reveal that one patient has greater residual muscle activity or reliance on synergistic patterns. There are numerous interventions available to stroke survivors, and EMG data may also inform treatment decisions. Certain interventions may be best suited for individuals with specific deficits detected from EMG (41). For all of these scenarios, extensive further research will be required to evaluate diagnostic and predictive value. These critical studies will determine whether the unique insights from EMG data justify the cost, training, and resources required to deploy these systems in acute care.

### Lesson 2: Navigating Complex Care Teams

**Figure 2:**
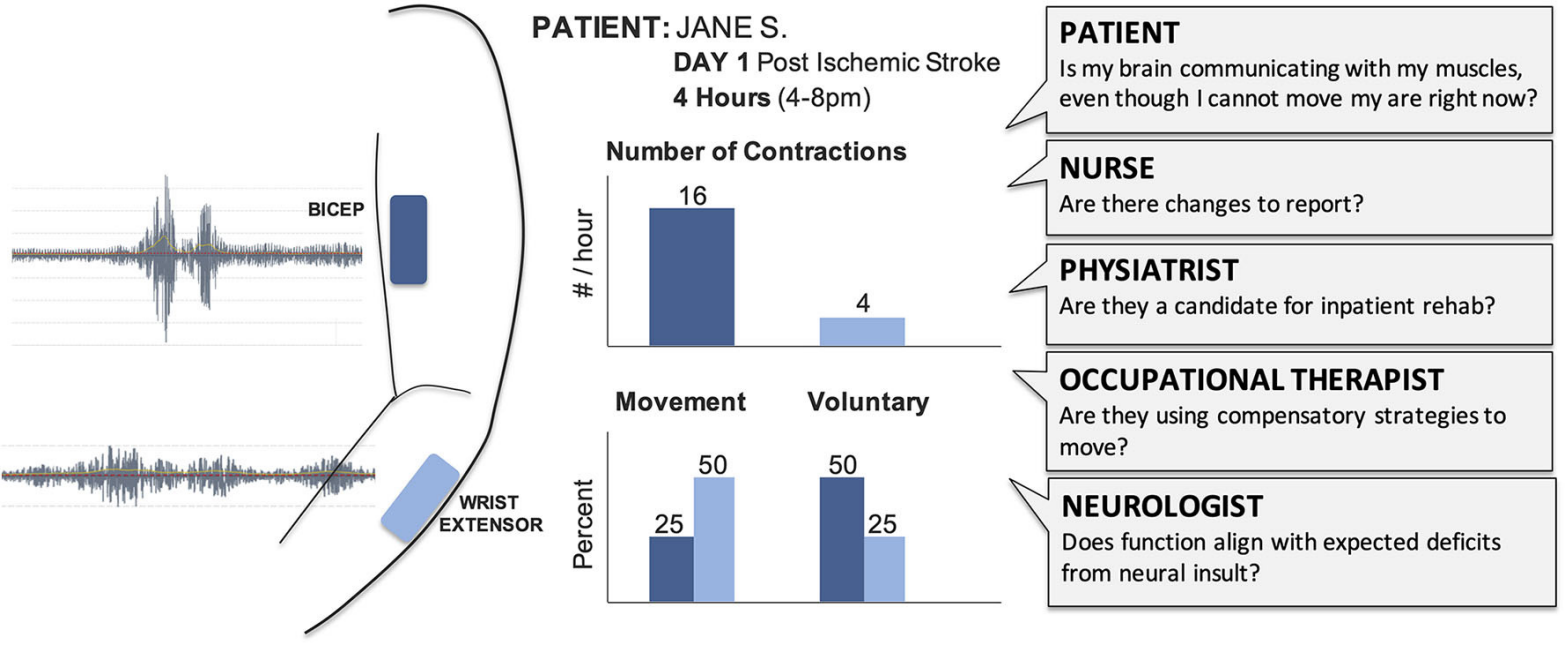
Raw EMG signals provide limited value for the clinical team and need to be transformed into summary results that can support care and treatment decisions. Summary sheets with clear and concise graphs and metrics support clinical discussions and documentation. Different clinicians will require different outcome metrics from EMG to address their specific questions. We have highlighted potential outcomes of interest for acute care, such as number of contractions and whether those contractions were voluntary or occurred with movement. For example, a physical therapist could view these hypothetical results to help them understand which muscles are demonstrating volitional control, select activities for their next session with the patient, and provide specific feedback to the patient and their family.

The large numbers of clinicians involved in acute care gives each patient a powerful team, but creates challenges for deploying new techniques, especially in time and resource-limited hospital settings (42). When introducing technology like EMG in acute care, we must ensure it will not interfere with existing tools and consider the potential insight offered for each team member (Figure 2). As EMG is typically not included in medical training (35), education would also be required. The frontline nurses would need to understand skin care and procedures (e.g., do these need to be removed before a shower?), while the physicians and therapists would need to understand how EMG data triangulates with other exams (e.g., does the presence of synergistic activity align with the injured brain regions?).

During our research, the physical and occupational therapists expressed the greatest interest in the EMG sensors and results. This likely reflects their prior exposure to muscle monitoring technologies (e.g., biofeedback and electrical stimulation), as well as the fact that much of their time is spent assessing movement. In the hospital where our data were collected, all patients participate in daily occupational and physical therapy sessions as soon as possible during acute care. The therapists were interested in which muscles were most active, and also if activity was present during therapy, particularly in patients with dense hemiplegia or emerging muscle strength. In considering who might deploy EMG in acute care, therapists may represent the best option although their current training involves very limited exposure to EMG. Preparing educational materials and ensuring EMG systems can be easily integrated into their care routines will be critical to support translation.

### Lesson 3: Preparing Round-Ready Results

It is not enough to just collect EMG data. The data also needs to be summarized and presented in a compelling form. We call these “round-ready results” —results that can be interpreted quickly, compared to prior days, evaluated relative to expected norms, integrated into standardized reports, and discussed by the team during clinical rounds or care conferences (43).

Creating a curated collection of results will require careful evaluation of the most relevant outcome measures and robust processing pipelines. Many different quantitative metrics can be evaluated from EMG data—such as number of contractions, contraction magnitude, contraction duration, presence of synergistic activations, evaluations of spasticity, or measures of voluntary vs. involuntary contractions (24, 25). For most applications, all of these metrics will not be necessary or desirable for a clinical team. Processing pipelines will be needed that not only calculate specific outcome measures from the raw EMG data that can be integrated into standardized reports, but also assist with set-up (e.g., providing reminders and simple instructions), evaluate signal quality (e.g., monitor background noise), and alert users if there are errors (e.g., detecting cross-talk). While we manually identified contractions, high quality sensors and new processing techniques can help automate many of the processing methods (44–47). Finding the balance between systems that minimize training time, while still giving clinicians confidence and flexibility will require intentional collaboration between rehabilitation engineers and clinicians. Engineers need to clearly demonstrate the possibilities of EMG to clinicians, while clinicians need to provide detailed feedback to develop useful, round-ready results.

### Lesson 4: Developing EMG Systems for Clinical Care

All of these applications require EMG systems that provide high quality data with easy-to-use, cost-effective, and comfortable interfaces. From our team's experience, there are currently no commercially-available EMG systems that are suitable for use in acute care settings. Research-grade systems provide high quality signals, but are often bulky and too costly for clinical use. Similarly, more-affordable systems often lack the specificity or signal quality to support clinical decision-making.

An ideal EMG system for use in acute care would be wireless and not require a base station, so that the patient can move freely within the clinic and minimize interference with other systems. The thin and flexible form factor of the BioStamp was excellent for use in acute care, but had significant technical limitations. The Bluetooth interface between the sensors and a tablet did not require additional equipment in the patient's room, but did increase the time for uploading and processing data. At only 0.3 cm thick, the sensors were comfortable to wear while lying in bed and had minimal interference with other activities. However, these sensors still required shaving and using adhesive and wrap to ensure the sensors did not fall off. The spacing between the electrode pairs was also too wide (48), which made it impossible to evaluate smaller muscles and increased the risk of crosstalk. We found that integration with an accelerometer or IMU was useful to evaluate whether contractions occurred with movement, although these additional sensors increase sensor size, decrease battery-life, and increase required memory storage.

An ideal sensor would use small, dry electrodes that eliminate the need to shave or use adhesives, yet can still target individual muscles and ensure high signal quality. Electrode arrays on cloth or other material that can flexibly fit around other equipment, intelligently identify active regions without precise alignment or consistent placement between sessions, may provide good options for clinical translation (49–53). Determining which muscles to monitor will also guide sensor development. Among stroke survivors, we found the wrist flexors and extensors provided some of the greatest differences between patients. Conversely, the triceps were consistently the most challenging to get high quality signals due to contact with the bed and skin and adipose tissues. Optimizing the EMG system, electrodes, and protocols to reduce burdens on clinicians and patients will be critical to create comfortable and flexible systems to support care and recovery.

## Conclusion

Our experiences in acute stroke care have highlighted the promise and challenges for using EMG data to evaluate muscle activity and enhance recovery. We are optimistic about the use of this technology in the clinic for stroke survivors. Our perspectives are drawn from deploying this technology during the first week after stroke in a well-resourced clinic in a major metropolitan hospital in the United States. To deploy EMG in other clinics will require careful consideration of the resources and needs of the clinic and patients. The development of EMG systems for stroke can also help accelerate the use of this technology in other areas, as clinicians gain greater experience and confidence with these techniques. Continuing to embed research in clinical environments will be a necessary prerequisite to translating EMG into standard care. Key questions still need to be addressed regarding the prognostic and diagnostic value from EMG monitoring. Biofeedback may provide a more immediate application to assist clinicians and patients in visualizing early muscle activity. Our team is excited by these future opportunities and confident that the current barriers can be overcome through collaborative efforts at the interface of engineering, rehabilitation, and data science.

## Ethics Statement

The studies involving human participants were reviewed and approved by the University of Washington Institutional Review Board. The patients/participants provided their written informed consent to participate in this study.

## Author Contributions

CP and HF collected the EMG data in the clinic. KS and CP analyzed the EMG data and wrote the first draft of the manuscript. All authors contributed to the manuscript revision, approved the submitted version, and to the conception of this perspective paper.

## Conflict of Interest

The authors declare that the research was conducted in the absence of any commercial or financial relationships that could be construed as a potential conflict of interest.
